# How Our Technology Use Changed in 2020: Perspectives From Three Youths

**DOI:** 10.2196/26154

**Published:** 2021-09-15

**Authors:** Babayosimi Fadiran, Jessica Lee, Jared Lemminger, Anna Jolliff

**Affiliations:** 1 West High School Madison, WI United States; 2 Dougherty Valley High School San Ramon, CA United States; 3 Chippewa Falls Senior High School Chippewa Falls, WI United States; 4 Department of Pediatrics University of Wisconsin - Madison Madison, WI United States

**Keywords:** mental health, social media, digital technology, youth, adolescent, commentary, technology, wellness

## Abstract

The Technology and Adolescent Mental Wellness program (TAM) is a research program with the primary goals of promoting research on the topic of adolescent technology use and mental wellness, creatively disseminating that research, and fostering community among stakeholders. Our foundational question is this: How can technology support adolescent mental wellness? Youth are key stakeholders in pursuit of this foundational question. In this commentary, we invited 3 members of TAM’s youth advisory board to respond to the following question: “How did your technology use change in 2020?” Jessica, Jared, and Babayosimi describe their technology use during COVID-19 as dynamic, and neither uniformly positive nor negative. Further, these 3 youths differ in their perceptions of the same technologies—social media and online school, for example—as well as their perceived ability to self-regulate use of those technologies. We invite you to weigh these perspectives just as we do at TAM—not as empirical findings in themselves, but as examples of youth ideas for future empirical investigation.

## Introduction

The Technology and Adolescent Mental Wellness program (TAM) is a national, United States–based research program with 3 primary goals. These goals include, first, to promote new research on adolescent technology use and mental wellness, through both project funding and the development of a data repository; second, to creatively disseminate that research through traditional academic means, such as the present theme issue, and nontraditional means, such as social media and industry-facing reports; and, third, to foster community and collaboration among diverse stakeholders. Our stakeholders include adult professionals at the intersection of youth, mental health, and digital technology, such as health care providers, therapists, educators, technology industry professionals, and parents. Furthermore—and perhaps most importantly—our stakeholders are youth themselves. The foundational question driving our program is this: How can technology promote adolescent mental wellness?

Youth are key stakeholders in pursuit of this foundational question. Recent systematic reviews on youth participatory action research (YPAR) [1-3] have evaluated the outcomes of engaging youth in “identifying, researching, and addressing social problems” [1]. These reviews find that youth engagement is associated with heightened youth agency, leadership, and the development of positive academic, social, and critical consciousness skills (eg, writing, belonging, and the ability to recognize social injustice) [1,3]. For communities, the benefit of youth engagement is just as positive; youth involvement in research has been associated with changes to organizational culture toward youth inclusivity and programs and policies that are more responsive to youth needs [2,3]. Importantly, however, one systematic review of YPAR found that youth are rarely involved in the early phases of the research process, such as needs assessment and the development of research questions [1]. Another systematic review found that none of the manuscripts were authored by youth [3]. In short, while the benefits of youth inclusion in academic processes are increasingly clear, the inclusion of youth as the authors of manuscripts and guides for the research agenda remains relatively unexplored.

There are key considerations when inviting youth to academic conversations. First, as investigators and scientists accustomed to seeking perfection in our own projects, papers, and processes, we must be receptive to adolescents’ developmental stage and degree of familiarity with academic processes. As with adults, writing abilities inevitably vary. And, as with adults, the availability of authorship opportunities for youth is not equally distributed; instead, it is a function of social determinants that are largely outside of youth’s control such as access to academic institutions or adult mentors. In this commentary, we do not claim to “fix” these issues, but merely to acknowledge them, and invite readers to consider for themselves if and how they could be addressed. We present the perspectives of 3 youths whose lives and, specifically, whose technology use were affected by the COVID-19 pandemic, including quarantine, virtual school, and social disruption. Our purpose is, first, to provide an example of empowering youth to be commentators in the academic space. Second, we hope that the perspectives shared will encourage others to include youth in every aspect of the research process—not just in the collection, interpretation, and dissemination of data, but also in the identification of unmet needs and catalysts of research questions.

In order to ensure that the TAM research program is responsive to youth needs, TAM is informed by a youth advisory board (YAB). Members of the TAM YAB are adolescents and young adults (ages 15-18) from around the United States who have a strong interest in the intersection between technology use and mental wellness. In November 2020, 3 members of the TAM YAB volunteered to contribute the following commentary to the academic conversation on adolescent technology use and mental health. Under the supervision of author AJ (who contributed the “Introduction” and “Discussion” sections), the youth authors—JaL, JeL, and BF—collaborated to identify a question of shared interest: “How did your technology use change in 2020?” The youth then created and revised their responses based on feedback from the theme issue co-editor and reviewers, and their final work is presented verbatim below.

## Jared Lemminger (Age 17, Grade 11)

In March 2020, the beginning of “quarantine” felt more like a vacation from school, but quickly became a period of boredom, loneliness, and oftentimes, frustration. Even with access to a seemingly infinite selection of entertainment and activities, these too became redundant. Once remote instruction finally started, it was very difficult to find the motivation to actually do it, as it was based on a work-at-your-own-pace platform, with a voluntary pacing guide and a deadline of June 1 to have everything completed. In September 2020, with the consent and direction of the county health department, my school district in Chippewa Falls, Wisconsin, began its first semester in-person, with various modifications to minimize the risk of COVID-19 transmission. Nearly 3 months in, we have, once again, temporarily transitioned back to a remote learning environment due to the incursion of COVID-19-related deaths in our community, with the intent to return to in-person learning once it is safe to do so.

Given this dynamic environment, I have gained insights into the effects these respective learning platforms have on my mental health. I can attest with absolute certainty that in-person learning has benefited my mental health substantially. Not only does it offer an opportunity for true camaraderie, but it also helps to restore a sense of normalcy amidst all that is uncertain about our modern world. I have found that while at home in remote learning, I wake up minutes before class begins, and conduct the rest of the day in Christmas-themed pajama pants, a graphic tee, and my retainer still tacked to my teeth. I can assure you that this is not a recommended habit. Most days, I would continually feel drained and unmotivated. This pattern of despondent behavior led to loneliness and diminished self-efficacy.

In addition, motivation to complete assignments and actively participate in class during remote learning has significantly diminished. Real-time remote instruction, for me, has been almost useless due to the fact that there is no obligation to engage, other than one’s own intrinsic motivation. The phrase “I will do it later” has become more prevalent while learning from home, when generally, I would consider myself a productive individual. This contributes to greater undue stress as I have to reteach myself that material through online resources and complete the accompanying assignments. I have fabricated a vicious cycle that can only be interrupted by solemnly attending class, but as I previously mentioned, that can only be achieved through intrinsic motivation (which I do not have).

Finally, the term “virtual” has become rather pervasive given that almost everything has become virtual. As a State Officer for a Career and Technical Student Organization, we have been forced to transition all in-person events and conferences to a virtual format. Our biggest obstacle has been discovering a level of engagement that continues to attract members to virtual events. I have a rather unique lens in this situation, because not only have I been experiencing the virtual fatigue myself, but I also have to plan appealing virtual conferences.

Interestingly enough, as a result of the increasingly virtual environment, my dependence on technology for entertainment purposes and social interaction has decreased. I less often feel pressure to be in constant communication and I do not seek satisfaction from superficial acquaintances. I have been much more authentic and have learned to live the way I am, and meant to be.

## Jessica Lee (Age 16, Grade 11)

When COVID-19 hit, I had no clue what technologies such as Zoom, Google Meet, and so many other amazing communication technologies were. The lockdown has forced me, like many others, to shift quickly. At first, it was a struggle to adapt—not to the increased technology use, but to the new technologies such as online conferencing and remote school that I had never truly experienced before. Similar to Jared’s experience, procrastination came easily to me. My sleep was affected because virtual school deadlines were often at midnight. I dealt with a lot of stress while adjusting. However, eventually, my mental and physical lifestyle benefitted from increased comfort with virtual technologies. Technology gave me the ability to multitask virtually anywhere. I could be taking a walk, and be learning about factoring polynomials at the same time. The versatility really improved my productivity in remote learning, and I quickly began to prefer being online.

However, with no mental break from technology, I also became much more aware of my constant use of technology. I had to begin shifting my phone usage to apps that were more beneficial to my mental health. Honestly, I still catch myself stuck and staring at my phone, feeling that I need to put it down and do something else, and being unable to even as I am beating myself up for it.

Around the beginning of the quarantine, one of my best friends went through an extremely rough time with her anxiety and depression, and I could not be there for her physically because of the lockdown. The only way I could communicate was through technology and texting, which can be known to not convey emotions well. It was really mentally taxing on me as I learned to interpret and understand what she was really trying to tell me. Because of this, I began having a more conscientious way of interacting with my friends through technology. I had to rethink how I communicated with those around me to be aware and considerate of how they might feel or interpret what I say. In-person, I could read people’s body language and tone of voice, I could offer a hug or nod of encouragement, but not in our current virtual age. Quarantine, social distancing, and my friends have helped me fathom just how much we all hide in text and how much those around me may be struggling.

I have now adapted my technology use so that I can better convey my emotions and understand those of others. I began spending less time staring at words and more looking at pictures, videos, memes or gifs, and my friend’s face through Zoom or FaceTime. Movement, sound, and motion seemed to reach people more than words. In my way of supporting my friend, I began something I like to call a Daily Tidbit: I send a meme, gif, or something I believe is worth sharing as a once-daily check-in. This was my way of saying, “I may not actually know what you are thinking, or how you feel now, but I will be there for you to stop those negative thoughts when they come.” These Daily Tidbits are how I virtually change her mindset and make her feel better. The things we do on our phones, behind our little screens, are important and they do have an impact.

## Babayosimi Fadiran (Age 17, Grade 12)

Unlike most teenagers, social media has not been an integral part of my life. I have had a smartphone since eighth grade but never downloaded a social media app until the tenth grade when my soccer team created a group chat on Snapchat to communicate practice and game information. However, when COVID-19 hit, most of my activities were canceled, leaving me isolated from close friends and bored at home for the summer of my senior year. I was left to look for other activities to do, including exploring the features on Snapchat. I soon learned how to view and create stories, which became part of my daily routine. Some of my friends have had multiple negative experiences with Snapchat due to political disagreements and high-school drama. However, for me, looking at stories showcasing my friends’ daily lives has made the limited human interaction bearable, and in fact more enjoyable in some cases.

In addition to using Snapchat, YouTube has become a bigger part of my life. Originally I only used YouTube to view entertaining satire or amazing feats. However, with quarantine, I started to “lose it” staying cooped up inside, so I decided on biking every day. But there was one problem: My bike required major repairs and I had little knowledge of the bike anatomy. Luckily, I could rely on YouTube to educate myself on lubrication, gear tension, and more. Ironically, YouTube caused me to get outside more.

Besides incorporating social media in my life during 2020, I also became accustomed to using video call apps, mostly Zoom. I am more of an introvert, so the first few Zoom school classes consisted of me turning off both my camera and sound. However, my Zoom habits changed when I had to switch from in-person cello lessons to virtual ones. Although the lag was troublesome for playing duets, I got more comfortable communicating via Zoom. I started leaving my camera on and contributing to school class discussions more. Similar to Snapchat, I explored the features Zoom had and used them to make video calls more engaging for others. For example, I am the president of a club called HOSA (Health Occupation Students of America) and have struggled to get others to engage in discussions. To combat this, we used the private chat of Zoom to play the game Taboo which has helped members get more comfortable speaking on Zoom.

Although quarantine has prevented me from participating in my normal activities during the year, it has also allowed me to discover new activities to engage in. In addition, it has helped learn the true capabilities of technology that I will be able to use in the future.

## Conclusion

“How did your technology use change in 2020?” The perspectives above represent just 3 possible responses to this question. Consistent with recent research on adolescent technology use during COVID-19, Jared, Jessica, and Babayosimi all experienced changes in their technology use in 2020 [4]. Their relationships with technology in 2020 were dynamic, neither uniformly positive nor negative, and the youth varied in both their perception of technology as having a positive or negative effect on their mental health, and their purported abilities to regulate their own technology use. Their personal accounts align with prepandemic findings, namely, that a population-level effect of technology use on mental health is difficult to detect [5]; that technology’s effects, positive of negative, may hinge on individual and social factors [6]; and that adolescents vary with respect to how well they regulate technology use [7]. We invite you to weigh these perspectives just as we do at TAM—not as empirical findings in themselves, but as examples of youth ideas for future empirical investigation.

## Abbreviations

HOSA: Health Occupation Students of America
TAM: Technology and Adolescent Mental Wellness program
YAB: youth advisory board
YPAR: youth participatory action research
